# Health care professionals’ knowledge of commonly used sedative, analgesic and neuromuscular drugs: A single center (Rambam Health Care Campus), prospective, observational survey

**DOI:** 10.1371/journal.pone.0227499

**Published:** 2020-01-10

**Authors:** Danny Epstein, Yaniv Steinfeld, Erez Marcusohn, Hanna Ammouri, Asaf Miller

**Affiliations:** 1 Department of Internal Medicine "B", Rambam Health Care Campus, Haifa, Israel; 2 Orthopedic Surgery Division, Rambam Health Care Campus, Haifa, Israel; 3 Medical Intensive Care Unit, Rambam Health Care Campus, Haifa, Israel; University of Rome 'La Sapienza', ITALY

## Abstract

**Background:**

Pain management and sedation are important aspects in the treatment of hospitalized patients, especially those mechanically ventilated. In many hospitals, such patients are treated not only in intensive care units, but also in other wards. In the nineteen eighties, numerous studies demonstrated a wide array of misconceptions and inadequate knowledge related to commonly used sedative, analgesics and muscle relaxants which may prevent appropriate treatment. Since these publications, multiple studies have shown that appropriate sedation and analgesia are associated with improved clinical outcomes, educational programs were developed and guidelines published. Whether the personnel’s knowledge kept up with these changes is unknown. The aim of this study was to determine the current rate of misconceptions and knowledge gaps regarding commonly used sedative, analgesic and neuromuscular drugs.

**Methods:**

In this prospective, observational, cross-sectional survey, a questionnaire was e-mailed to physicians and nurses routinely treating mechanically ventilated patients in Rambam Health Care Campus (Haifa, Israel).

**Results:**

355 questionnaires were returned. 82.54% knew that midazolam has no analgesic effect. 71–72% were familiar with the sedative effect of opiates. 27% believed that propofol has analgesic properties and 30.52% thought that rocuronium has a sedative effect.

**Conclusion:**

Our findings demonstrate that although a lot has been done during the last decades in order to improve the treatment of critically ill patients, the rate of misconceptions regarding pharmacological characteristics of commonly used drugs is unacceptably high. We call for performance of similar surveys in other institutes and for immediate action to improve patients’ care.

## Introduction

Adequate pain management and sedation are important aspects of treating critically ill patients. These necessitate the use of validated assessment tools in combination with multidisciplinary management approach and are associated with improved outcomes, reduced duration of mechanical ventilation, reduced infection rate, decrease in the length of hospital stay and 30-day mortality.[1] Despite the publication of numerous studies and guidelines dealing with sedation and analgesia in ICU, nearly 50% of patients report moderate to severe pain during common procedures performed throughout their hospitalization.[1–3] These procedures, commonly underappreciated as painful by the medical staff, include tracheal suctioning, mobilization and dressing changes. Immobility itself may cause muscle stiffness and pressure ulcers.[1] As much as 50% of patients discharged from the ICU remember the pain as their worst experience.[4] 30–80% of postoperative patients experience moderate to severe pain during hospitalization, which often continues long after discharge from hospital. [5] Unrelieved pain may contribute to the development of post-traumatic stress disorder, persistent pain and poor quality of life.[6]

While appropriate sedation and analgesia may pose its own challenge, the medical staff's knowledge may create additional obstacles and hinder appropriate treatment. Inadequate knowledge and misconceptions related to characteristics and appropriate dosages of sedative, analgesics and muscle relaxants may be one of these factors. Numerous studies published in the 1980’s, found that a significant number of critically ill, mechanically ventilated patients might have been treated with muscular blockage agents without concomitant narcotics or sedatives.[7,8] In 1989, Loper at al. surveyed the pharmacological knowledge of ICU physicians and nurses regarding the analgesic and anxiolytic effects of narcotics, benzodiazepines and neuromuscular blockers.[9] The results of their work demonstrated how common these misconceptions are. The findings of these trials are cited in different textbooks and in more than 80 studies.[10] Since the publication of these worrisome findings, educational programs have been developed, guidelines were published, and new agents were integrated into clinical practice.[11] The effect of these changes on the clinical knowledge of house-staff, and the rate of misconceptions are unknown.

In order to estimate the level of knowledge and misconceptions rate, we conducted a survey among physicians and nurses working in a tertiary academic hospital, regarding the pharmacological characteristics of commonly used sedative, analgesic and neuromuscular blocking agents.

## Methods

The study was approved by the Rambam Health Care Campus (RHCC) Institutional Review Board (approval number RMB-0470-18). The requirement for written informed consent was waived by the Institutional Review Board.

### Study design

This is a prospective observational cross-sectional study, using an electronically mailed anonymous self-administered survey, targeting physicians and nurses working in RHCC in Haifa, Israel.

A web-based 20 question questionnaire was designed by the authors to investigate the clinical knowledge of physicians and nurses regarding the pharmacology of commonly used sedative, analgesic and musculoskeletal blocking agents. The questionnaire was based on previously published and extensively cited survey performed by Loper et al. in 1989 with appropriate updates. [9] The survey was designed according to the BRUSO acronym (Brief, Relevant, Unambiguous, Specific, and Objective) published by Robert A. Peterson. [12] The digital survey included three sections:

1. A short explanation regarding the study rationale and a clarification that the participation in the study is purely voluntary and anonymous;
2. Demographic questions regarding the age, gender, profession (nurse or physician), experience, seniority (only for physicians) and department where the participant is employed. In addition, each responder was asked if he is routinely treating mechanically ventilated patients.
3. Knowledge section- the participants were asked only two short questions regarding five sedative, analgesic and musculoskeletal blocking agents- does the agent have a sedative and analgesic characteristics, for example: “Does Midazolam have an analgesic effect?”. For each question there were four possible answers: “Yes”, “No”, “I do not know” and “I do not use this agent”.

The complete questionnaire can be found in S1 File.

The RHCC is a 1000-bed academic hospital, serving a population of over two million residents in northern Israel, with 80,000–90,000 hospitalizations annually. In RHCC mechanically ventilated patients are hospitalized in 5 ICUs (surgical, medical, cardiac, cardio thoracic and neurosurgical), 5 internal medicine and 2 general surgery wards. In addition, mechanically ventilated patients are treated in the Emergency Room (ER) for up to 48 hours. ICUs are staffed by intensive care physicians, cardiologists, neurosurgeons, and thoracic surgeons, during the day shifts, and by anesthesiology, surgery (general, thoracic, neuro-surgery and orthopedics), and internal medicine residents, during the night shifts. Intravenous sedatives, analgesics and neuromuscular blockers are allowed and used, as indicated, in all the departments treating mechanically ventilated patients.

During December 2018, a link to the digital questionnaire and a cover letter was e-mailed to 426 physicians and 579 nurses working in medical and surgical departments where mechanically ventilated patients are treated. Non-responders were sent the link again via phone text message. The participation was purely voluntary, and by completing the survey it was implied that consent has been given to participate in the study. The surveys were filled out anonymously. The study population was composed of physicians and nurses who electronically submitted the completed forms. Only participants working in medical and surgical wards treating mechanically ventilated adult patients were included in the survey analysis.

### Statistical analysis

We calculated that a sample size of 278 participants (while the entire population is 1005) would provide 95% confidence interval and 5% margin of error. Recent surveys in anesthesiology reported 54 to 33% response rates. [13]

Baseline characteristics were summarized using descriptive statistics. The Kolmogorov–Smirnov test was used to examine significant deviations from the normal distribution for continuous variables. Student’s t-test for independent samples was used to compare means of variables with normal distribution, and Mann-Whitney U test was used for variables that deviated from the normal distribution. Chi-squared test and Fisher exact test were used to analyze differences between dichotomous variables, depending on the sample size. P-value<0.05 was considered statistically significant.

Data analysis was conducted with Statistical Package for the Social Sciences, version 23.0 (SPSS, Chicago, IL, USA), Microsoft Excel version 14.0 (Microsoft Corporation, Redmond, Washington) and with MedCalc Statistical Software version 14.8 (MedCalc Software, Ostend, Belgium).

This manuscript adheres to the applicable Strengthening the Reporting of Observational studies in Epidemiology (STROBE) guidelines.

## Results

After two attempts of communication (e-mail and SMS), 404 questionnaires were returned. 49 responders were excluded from the analysis because they were not treating mechanically ventilated patients on a regular basis. Three hundred fifty five questionnaires (87.87%) were included in the study.

The response rate was 42.49% (181/ 426) for physicians and 30.05% (174/ 579) for nurses. Participants’ characteristics are summarized in Table 1.

**Table 1:** Demographic characteristics of 355 physicians and nurses who returned the questionnaires and were included in the study. Values are mean (±Standard Deviation), median (interquartile range [range]) or number (percent %).

		Total (n = 355)	Physicians (n = 181)	Nurses (n = 174)
**Age**- years*	Mean ±SD	39.26 ± 9.68	36.56 ± 9.12	42.11 ± 9.45
	Median (IQR)	37 (32–46)	34 (31–39)	42 (35–49)
**Male sex**- no (%)**		200 (56.66%)	134 (74.03%)	66 (37.93%)
Experience***	Less than 5 years- no (%)	119 (34%)	100 (55.87%)	19 (11.11%)
	Between 5 to 10 years- no (%)	70 (20%)	35 (19.55%)	35 (20.47%)
	More than 10 years- no (%)	161 (46%)	44 (24.58%)	117 (68.42%)
**Seniority**^#^	Resident- no (%)	-	52.66%	-
	Specialist- no (%)	-	34.32%	-
	Other- no (%)	-	13.02%	-
**Clinical department**^##^	Intensive care- no (%)	48 (13.52%)	6 (3.35%)	42 (24.7%)
	Anesthesiology—no (%)	31 (8.73%)	21 (11.73%)	10 (5.88%)
	Emergency care department- no (%)	40 (11.27%)	10 (5.59%)	30 (17.65%)
	Internal medicine division- no (%)	126 (35.49%)	60 (33.52%)	64 (37.65%)
	Surgical division- no (%)	68 (19.15%)	51 (28.49%)	17 (10%)
	Other- no (%)	38 (10.7%)	31 (17.32%)	7 (4.12%)

* Data regarding age was available for 337 responders (94.93%).

** Data regarding sex was available for 353 responders (99.44%).

*** Data regarding experience was available for 350 responders (98.59%).

^#^ Data regarding seniority was collected only for physicians only and was available for 169 responders (93.37%).

^##^ Data regarding specialty was available for 351 responders (98.87%).

SD- Standard Deviation; IQR- interquartile range.

**Fig 1:**
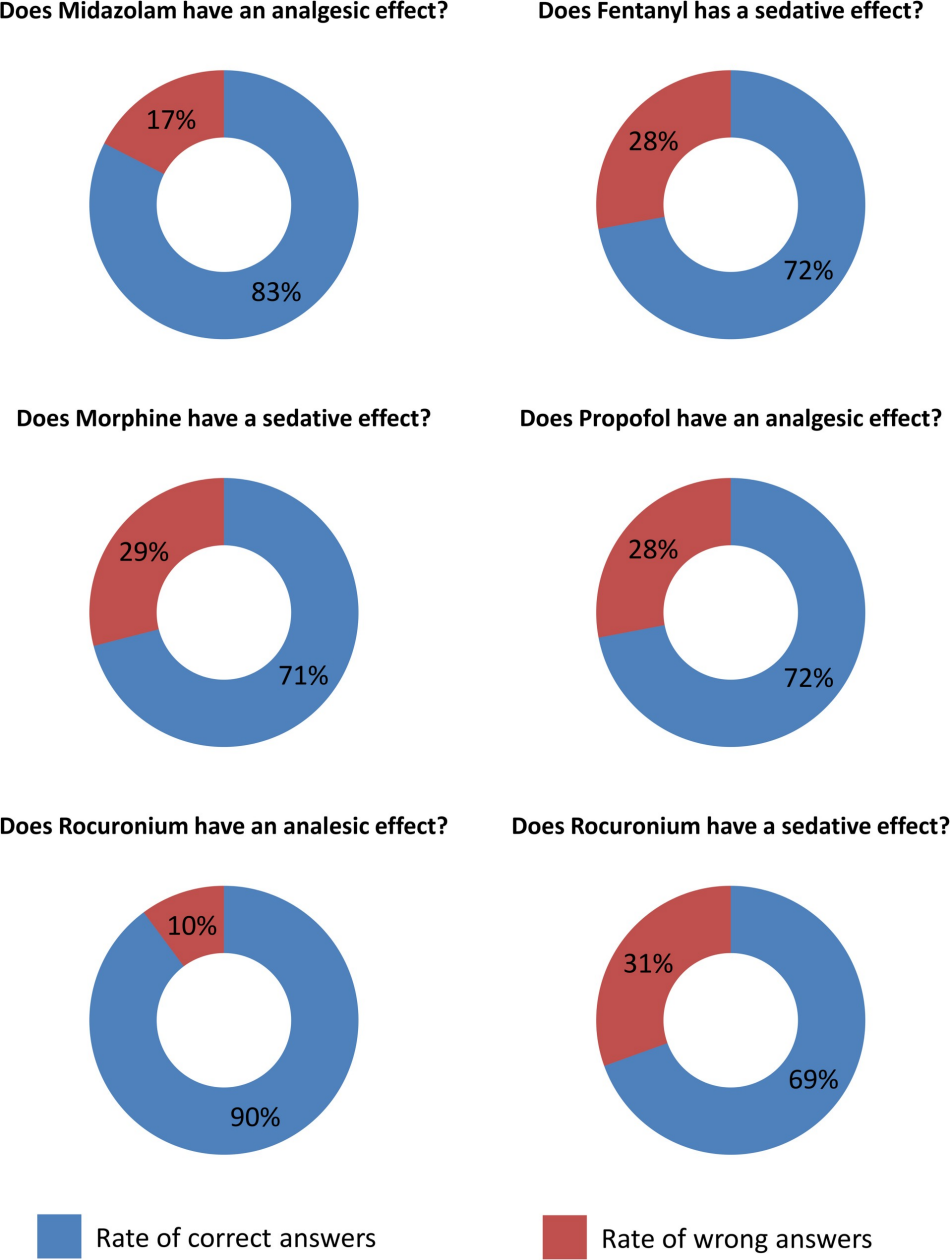
The rate of correct answers to main questions in the survey (n = 355).

The main survey’s findings are presented in Fig 1.

### Midazolam

All responders declared they are familiar with midazolam (n = 355). Although midazolam has no analgesic effect, only 82.54% (n = 293) of responders recognized it as a solely sedative medication. The other 17.46% believed it has an analgesic effect as well. The rate of correct response was slightly higher among nurses than among physicians (86.78% vs. 79.01%), the difference was not statistically significant (p = 0.07). 87.5% and 95% of ICU and ER staff, respectively, recognized the pharmacological effect of midazolam correctly. The rate of correct answers was significantly higher among ER and ICU staff compared to other departments (90.91% vs. 79.85%, p = 0.02). Significantly more caregivers with clinical experience of less than five years were wrong regarding the anesthetic effect of midazolam compared to their more experienced colleagues (73.11% vs. 87.01%, p = 0.002). Among physicians, significantly more specialists were aware of the pharmacological effect of midazolam than their younger colleagues (89.66% vs. 74.77%, p = 0.03).

### Fentanyl and morphine

All the responders declared they were familiar with fentanyl and morphine (n = 355). Although 96.34% and 100% of the responders, respectively, knew these drugs have an analgesic effect, only 72.11% and 70.99%, respectively, were familiar with their sedative effect. The rate of correct responses was not significantly different between physicians and nurses (89.5% vs74.71%, p = 0.34 and 71.82% vs. 69.54%, p = 0.72, respectively). 73.81% and 63.33% of ICU and ER staff correctly recognized the sedative effect of fentanyl, respectively. 73.81% and 53.33% of ICU and ER staff correctly recognized this pharmacological effect of morphine, respectively. The knowledge regarding the sedative effect of opiates was similar among ICU staff and other wards’ staff (71.29% for fentanyl and 69.3% for morphine, p = 0.87 and 0.7, respectively).

Less experienced caregivers (with less than five years of experience) had significantly higher rate of misconceptions regarding the sedative effect of fentanyl compared to those with more experience (63.03% vs. 76.62%, p = 0.01).The differences among residents and senior physicians were not statistically significant (70.27% vs. 82.76%, p = 0.11 for fentanyl and 77.59% vs. 76.58% for morphine, p = 0.96, respectively).

### Propofol

All responders were familiar with propofol (n = 355). 27% (n = 98) assumed it has analgesic properties. Significantly more nurses than doctors (82.18% vs. 64.09%, p = 0.0002) recognized it as a purelysedative medication. 85.42% and 82.5% of ICU and ER staff, respectively, correctly recognized the solely sedative effect of propofol. Significantly less caregivers working outside ICU and ER were aware of the solely sedative effect of propofol, (84.09% vs. 69.29%, p = 0.01).

We found no significant association between the experience of caregivers and knowledge of the properties of propofol (67.23% for those with experience of less than five years and 75.32% for more experienced house staff, p = 0.07). The differences among residents and senior physicians were not statistically significant, (63.79% vs.71.17%, p = 0.42).

### Rocuronium

Three hundred forty four (96.9%) responders were acquainted with rocuronium and using it on a regular basis. 89.83% of the responders were aware of the fact that rocuronium has no analgesic effect. However, only 69.48% of the responders knew that this neuromuscular blocker has no sedative effect. 88.41% of nurses and 91.11% of physicians correctly recognized the lack of analgetic effect of rocuronium (p = 0.52). Significantly more physicians were aware of the lack of sedative effect of this agent compared to nurses, 80.56% vs. 57.32%, p<0.0001.

The majority of ICU and ER staff, (93.75% and 92.5% respectively), answered correctly regarding the lack of analgesic effect of rocuronium, while only 83.33% and 60% of ICU and ER staff, respectively, knew it has no sedative effect. Among medical personnel outside the ICU, 88.59% and 66.78% correctly answered the questions regarding analgesic and sedative effect of the drug, respectively. Although the findings regarding the analgesic effect of rocuronium were not statistically different (p = 0.4), significantly more caregivers in the ICU compared to other wards were aware of the lack of sedative effect of this agent (p = 0.033). We found no significant association between the experience of caregiver and the knowledge regarding the analgesic effect of rocuronium (88.24% for those with less than five years of experience vs. 86.15% for those with more experience, p = 0.26). Significantly less house staff with more than ten years of experience correctly recognized the lack of sedative effect of this agent, 62.32% vs. 75.63%, p = 0.029. The differences between residents and senior physicians were not statistically significant: 93.1% of seniors correctly recognized the lack of analgesic effect vs. 89.19% of residents (p = 0.58) and 82.76% of seniors correctly recognized the lack of sedative effect vs. 76.58% of residents (p = 0.46).

### Total score

**Fig 2:**
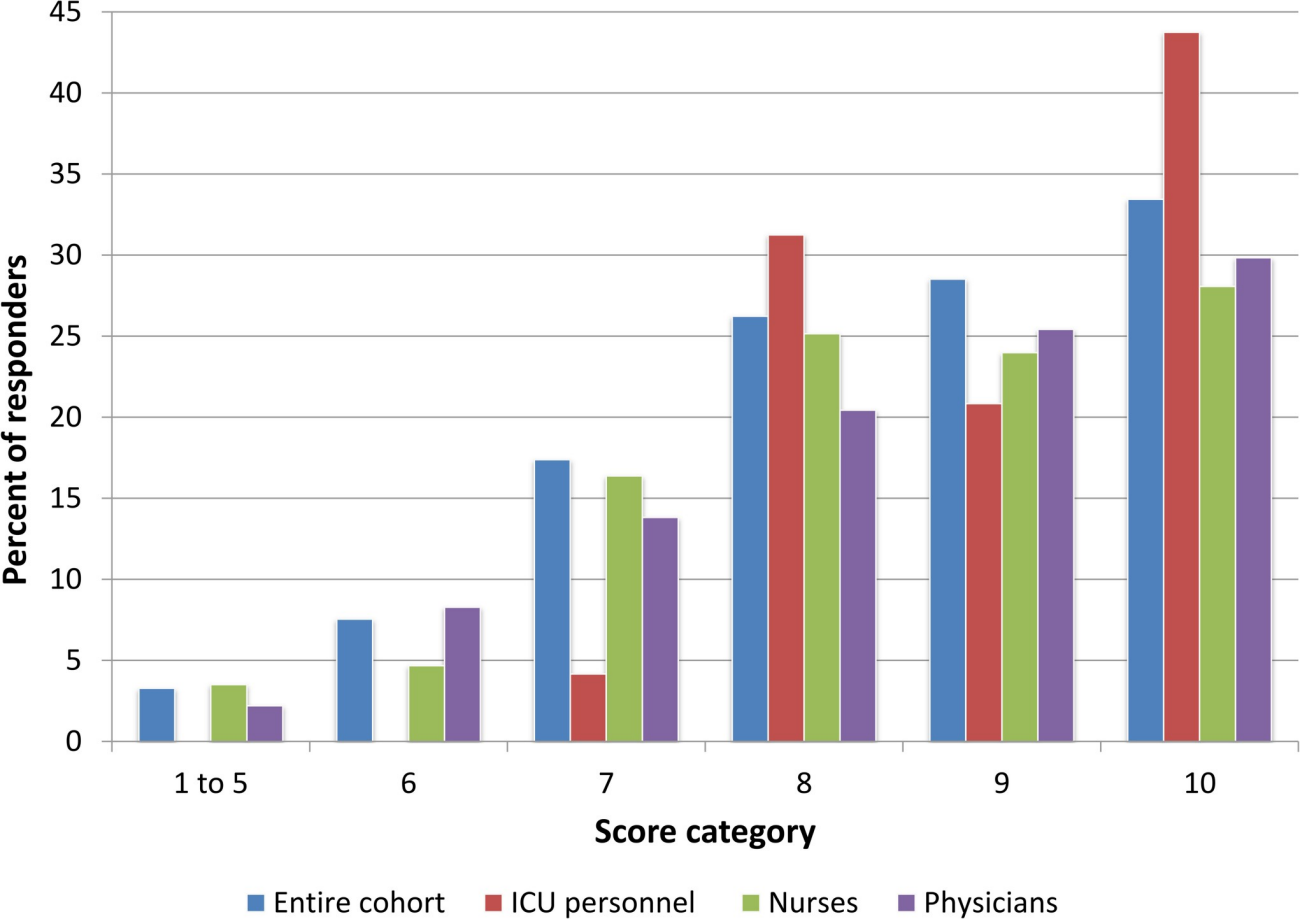
Giving each question one point (two questions for each agent were included in the survey, one regarding the analgesic effect and the second regarding sedative effect), a score on a scale from zero to ten for each questionnaire was generated. Scores’ distribution of 355 questionnaires included in the analysis, the physicians’ (181) and nurses’ (174) scores, as well as the scores of ICU staff (48) are shown. Low scores (1–5) are presented together.

Giving each question one point (two questions for each agent were included in the survey, one regarding the analgesic effect and the second regarding sedative effect), we generated a score on scale from zero to ten for each questionnaire. The median score for the entire population was nine (interquartile range (IQR) 8–10). There were no differences between physicians and nurses, median score of 9 (IQR 8–10) for both, p = 0.66. Caregivers with experience of at least five years scored significantly higher than their younger colleagues, median score was nine (IQR 8–10) vs. eight (IQR 7–9), p = 0.045. ICU personnel and caregivers working outside ICU departments both scored nine with a different IQR (8–10 vs. 7–10), reaching statistical significance, p = 0.002. Among physicians, seniors scored better than residents with median score of nine (IQR 8–10) vs. 8 (IQR 7–9), p = 0.006. Additional information regarding scores distribution is shown in Fig 2.

## Discussion

This single center, prospective, observational survey showed a high rate of misconceptions regarding pharmacological characteristics of commonly used drugs sedative, analgesic and neuromuscular drugs among medical personnel.

Frequently cited studies conducted a few decades ago, found that a large portion of medical personnel working in ICU held the misconception that benzodiazepines provide analgesia and that neuromuscular blockers were both sedative and analgesic. The authors concluded that this situation is unacceptable and raised the alarming possibility that ventilated patients may be paralyzed but aware and suffering from pain. Immediate implementation of educational programs was recommended. [7–9] While in the 1980s and 1990s deep sedation and neuromuscular blockade were the common practice, newer guidelines emphasize the analgesia-first concept, the benefits resulting from a lighter level of sedation and the importance to avoid unnecessary muscular blockade.[14] Another important factor influencing current clinical practice is the fact that in many hospitals mechanically ventilated patients are not managed exclusively in the ICU, but also in surgical and medical wards. [15] Even those physicians and nurses who do not treat mechanically ventilated patients on a regular basis, deal with sedated patients and parenteral analgesia in orthopedic wards, burn units, ERs, and other wards.[16] In this reality, we expect the medical staff of tertiary academic hospitals to be familiar with the pharmaceutical effect of commonly used sedatives, analgesics, and muscle relaxants.

Unfortunately, the findings of our study reflect a different and worrisome reality highlighted by high rate of misconceptions among a wide range of medical specialties.

In our survey, we found that most medical personnel were familiar with the sedative effects of midazolam and propofol, which is becoming the main sedative medication in ICU today.[3] However, almost a third thought that these drugs pose analgesic effects as well. As for opiates, although they were considered to have analgesic properties in almost all questionnaires, a large proportion was unaware that they have a sedative effect.

These basic misconceptions are in contrary with the guidelines recommending an analgesia- first sedation strategy and may lead medical personnel to provide excessively deep sedation and lack of analgesia on one hand, and to treat patients receiving opiates with unnecessary midazolam or propofol, on the other hand. Such nonessential interventions may cause hemodynamic instability, prolong ventilation and hospitalization, induce delirium and lead to a variety of complications during sedation of spontaneously breathing patients.[3]

Rocuronium is a non-depolarizing neuromuscular blocking agent that has no analgesic or sedative effects. However, almost a third of all responders believed it to have sedative properties, and more than ten percent considered it to have an analgesic effect. As much as 40% of emergency room personnel and 17% of ICU staff believed that muscle relaxants have a sedative effect. Using muscle relaxants without concomitant sedatives or analgesics may cause a situation in which patients are aware of their surroundings and feel pain without having the ability to express it. This terrifying scenario has been the subject of descriptive non-medical articles and movies. It is one of the biggest fears people have when they are about to undergo a medical procedure.[7,17]

The misconception rate regarding the pharmacologic effects of benzodiazepines and propofol was higher among physicians, compared to nurses, while physicians had better knowledge about opiates and muscle relaxants. Senior physicians scored better than their younger colleagues. The fact that more experienced care givers had higher rate of misconceptions regarding sedative effect of muscle relaxants, may reflect an outdated practice of liberal and PRN muscle relaxants administration. Although ICU medical personnel scored better in all the parameters, we believe that the rate of misconceptions among this population is unacceptably high.

Our findings call for performance of similar surveys in other institutes and for immediate action to improve patients’ care. There is no doubt that personalized educational programs addressing specific knowledge gaps of each population and periodic obligatory exams are necessary as well as incorporation of safe sedation and analgesia courses into the syllabus of any medical and surgical residency training. However, the fact that the same knowledge deficits present for more than 30 years raise the possibility that more rigid “safeguards” should be employed. These may include elaboration of sedation and analgesia protocols in all departments treating mechanically ventilated patients as well as daily clinical pharmacist review of sedatives, analgesics and paralytics administered. In different fields of medicine, including intensive care, strong evidence supports the use of clinical decision support tools to achieve improvement in care delivery and adherence to medical guidelines.[18,19] Incorporation of automated clinical decision support tools into an electronic health record system of mechanically ventilated patients may assist caregivers in correct selection of medications and may prevent insufficient or excessive sedation and analgesia.

Our study has some limitations. Being a single center study, its findings may be difficult to generalize to other medical facilities, although the physicians and nurses working in RHCC graduated from different faculties in Israel and abroad, increasing the chance that our findings reflect a more widespread problem. The findings of this study cannot be generalized to centers where ventilated patients are treated solely in ICUs by much smaller and selective group of caregivers. However, we believe that in many countries ventilated patients are treated in variety of medical wards outside ICU. The response rate in our study was 35.32% and it is close to response rates reported in other studies in anesthesiology. [20,21] The response rates in surveys in other fields ranges between 4 to 64%. [22,23] Although there is no agreement in the literature on a minimum acceptable response rate, it ranges between 30 to 50%. [13,24] The response rate in our study may make the results a subject to “nonresponse bias”. [13] The participation in the survey was voluntary, therefore we believe that most of the responders believed they knew the correct answers. We assume that making the participation obligatory will increase the rate of false answers. Dexmedetomidine was not included in our study because this agent is not available in most ICUs in Israel.

In summary, this study emphasizes the unsatisfactory situation among nurses and physicians treating critically ill patients who need sedation and analgesia. In this reality, it seems crucial to promote educational programs and elaborate protocols and guidelines in the ICU, and other medical and surgical wards.

## Supporting information

S1 File Questionnaire emailed to the participants (English and Hebrew versions). (DOCX)
