# Supplementary material for: Health care professionals’ knowledge of commonly used sedative, analgesic and neuromuscular drugs: A single center (Rambam Health Care Campus), prospective, observational survey
Source: PLoS One. 2020 Jan 10;15(1):e0227499. doi: 10.1371/journal.pone.0227499 (PMC6953819; doi:10.1371/journal.pone.0227499)
Supplement: S1 File — (DOCX) [file pone.0227499.s001.docx]

**Questionnaire- English Version**

|  | **Optional answers** | **Question** |
| --- | --- | --- |
| **Study rationale and informed consent to participate** | | |
|  | - I agree to take part in the survey - I DO NOT agree to take part in the survey | You are invited to participate in a web-based online survey regarding commonly used sedative, analgesic and neuromuscular drugs.  Your participation in this survey is completely voluntary. You may refuse to take part in the research or exit the survey at any time without penalty. You are free to decline to answer any particular question you do not wish to answer for any reason.  We do not collect any identifying information such as your name, email address, or IP address.  Please answer the following questions as honestly as possible without using literature or websites. |
| **Demographic information** | | |
|  | - Male - Female | What is your gender? |
|  | ________________ | Please type your age in years |
|  | - Physician - Nurse | What is your profession? |
|  | - 1 to 5 years - 5 to 10 years - More than 10 years | How many years of experience do you have? |
|  | - Resident - Specialist | Are you resident or specialist (only for physicians)? |
|  | ________________ | Please type the department where you work |
|  | - Yes - No | Do you treat mechanically ventilated patients on daily basis? |
| **Knowledge section** | | |
|  | - Yes - No - I do not know - I do not use this drug | Does Midazolam (Dormicum) have an analgesic effect? |
|  | - Yes - No - I do not know - I do not use this drug | Does Midazolam (Dormicum) have a sedative effect? |
|  | - Yes - No - I do not know - I do not use this drug | Does Fentanyl have an analgesic effect? |
|  | - Yes - No - I do not know - I do not use this drug | Does Fentanyl have a sedative effect? |
|  | - Yes - No - I do not know - I do not use this drug | Does Morphine have an analgesic effect? |
|  | - Yes - No - I do not know - I do not use this drug | Does Morphine have a sedative effect? |
|  | - Yes - No - I do not know - I do not use this drug | Does Propofol have an analgesic effect? |
|  | - Yes - No - I do not know - I do not use this drug | Does Propofol have a sedative effect? |
|  | - Yes - No - I do not know - I do not use this drug | Does Rocuronium (Esmeron) have an analgesic effect? |
|  | - Yes - No - I do not know - I do not use this drug | Does Does Rocuronium (Esmeron) have a sedative effect? |

**Questionnaire- Original (Hebrew) Version**

|  | **שאלה** | **אופציות תשובה** |
| --- | --- | --- |
| הסכמה מדעת להשתתף בסקר | | |
|  | לפניך סקר קצר בנושא תכונות התרופות השונות הניתנות לרוב לחולים מונשמים בעת אשפוזם בבית החולים.  נודה לך אם תסכים/י לענות על שאלון קצר בנושא. ההשתתפות בסקר הנה התנדבותית. השאלון הנו אנונימי ובשום שלב לא תתבקש/י למסור שם או פרטים מזהים אחרים. איננו אוספים אף פרט מזהה במהלך התהליך.  נבקש להשיב על השאלות בהתאם לידע הקודם שיש לך בנושא מבלי להיעזר בספרות או אתרי אינטרנט. | 1. אני מוכן להשתתף בסקר 2. אינני מוכן להשתתף בסקר |
| פרטים דמוגרפיים- אנא ציין את הפרטים הדמוגרפיים הבאים: | | |
|  | מין | 1. זכר 2. נקבה |
|  | גיל | בחירה מרובה בין 18-70 שנים |
|  | תחום העיסוק | 1. רופא 2. אחות |
|  | ותק במקצוע | 1. 1 עד 5 שנים 2. 5 עד 10 שנים 3. מעל 10 שנים |
|  | במידה והנך רופא, אנא ציין את השלב המקצועי | 1. מתמחה 2. מומחה |
|  | באיזו מחלקה הנך עובד? | שאלה פתוחה |
|  | האם הנך מטפל בחולים מונשמים כחלק מהעיסוק היומיומי? | 1. כן 2. לא |
| שאלות ידע | | |
|  | האם ל-Midazolam (Dormicum) ישנן תכונות משככות כאב? | 1. כן 2. לא 3. לא יודע 4. אינני מכיר את התרופה |
|  | האם ל-Midazolam (Dormicum) ישנן תכונות סדטיביות? | 1. כן 2. לא 3. לא יודע 4. אינני מכיר את התרופה |
|  | האם ל-Fentanyl ישנן תכונות משככות כאב? | 1. כן 2. לא 3. לא יודע 4. אינני מכיר את התרופה |
|  | האם ל-Fentanyl ישנן תכונות סדטיביות? | 1. כן 2. לא 3. לא יודע 4. אינני מכיר את התרופה |
|  | האם ל-Morphine ישנן תכונות משככות כאב? | 1. כן 2. לא 3. לא יודע 4. אינני מכיר את התרופה |
|  | האם ל- Morphine ישנן תכונות סדטיביות? | 1. כן 2. לא 3. לא יודע 4. אינני מכיר את התרופה |
|  | האם ל-Propofol ישנן תכונות סדטיביות? | 1. כן 2. לא 3. לא יודע 4. אינני מכיר את התרופה |
|  | האם ל-Propofol ישנן תכונות משככות כאב? | 1. כן 2. לא 3. לא יודע 4. אינני מכיר את התרופה |
|  | האם ל- Rocuronium(Esmeron) ישנן תכונות סדטיביות? | 1. כן 2. לא 3. לא יודע 4. אינני מכיר את התרופה |
|  | האם ל- Rocuronium(Esmeron) ישנן תכונות משככות כאב? | 1. כן 2. לא 3. לא יודע 4. אינני מכיר את התרופה |
